# Electrochemical Sensor Based on Laser-Induced Graphene for Cortisol Detection in Physiological Media

**DOI:** 10.3390/bios16100562

**Published:** 2026-10-05

**Authors:** Shuai Zhang, Chao Zhang, Jingwei Zhang, Yuran Wang, Yingze Sun, Yijiang Zhou, Fengnian Zhao

**Affiliations:** 1 Information Technology Research Center, Chinese Academy of Fishery Sciences, Beijing 100141, China; 2431042212@st.btbu.edu.cn (S.Z.); zhangchao@cafs.ac.cn (C.Z.); sunyz@cafs.ac.cn (Y.S.); 2 School of Light Industry Science and Engineering, Beijing Technology and Business University, Beijing 102488, China; 2531042282@st.btbu.edu.cn (J.Z.); 2531041104@st.btbu.edu.cn (Y.W.); beijing20300302@163.com (Y.Z.)

**Keywords:** cortisol, laser-induced graphene, gold nanoparticles, Nafion, electrochemical detection

## Abstract

As a key biomarker for regulating stress levels, timely detection of cortisol can provide an immediate indication of a person’s current stress status and serve as a valuable reference for preventing related diseases. In this study, a laser-induced graphene (LIG)-based electrochemical sensor for rapid cortisol detection was developed. The prepared LIG/Au/Nafion interface exhibits higher electrocatalytic activity toward cortisol than the LIG/Au interface, attributed to the sulfonate-rich interfacial microenvironment of Nafion, which facilitates the interfacial conduction and removal of the protons liberated during cortisol dehydrogenation. Under optimal conditions, the obtained sensor shows a good linear relationship for detecting cortisol solutions over a wide linear range (20–2000 nM) and a low detection limit (1.02 nM). Furthermore, the sensor exhibited a good linear relationship with artificial sweat samples, and the recovery rate for cortisol in actual sweat samples ranged from 96.76% to 113.14%, offering a promising new technical pathway for rapid detection of cortisol in physiological media.

## 1. Introduction

Cortisol is a glucocorticoid hormone primarily synthesized and secreted by the zona fasciculata of the adrenal cortex. After being transported to various tissues throughout the body via the bloodstream, it can exert physiological effects, including regulation of blood pressure [1], suppression of inflammatory responses [2,3], regulation of the immune system [4], and modulation of stress responses [5]. Excessive cortisol secretion may lead to emotional changes such as anxiety and depression [6]. Conversely, insufficient cortisol secretion may trigger conditions such as primary adrenal insufficiency [7]. Furthermore, prolonged exposure to stressful environments can increase cortisol production, leading to issues such as Cushing’s syndrome [8], an elevated risk of endometrial cancer [9], and post-traumatic stress disorder [10]. Therefore, establishing a method to promptly and accurately measure cortisol levels in the human body is crucial for understanding an individual’s health status, emotional state, metabolism, and immune response.

Currently, a variety of methods have been applied for cortisol detection, including liquid chromatography–tandem mass spectrometry (LC-MS/MS) [11], enzyme-linked immunosorbent assay (ELISA) [12], and radioimmunoassay (RIA) [13]. It should be emphasized that, while LC-MS/MS is known for its high specificity, it is limited by the high cost of equipment, cumbersome sample preparation procedures, and the need for highly skilled operators [14], making it difficult to meet the demands of rapid on-site testing. Furthermore, although ELISA and RIA offer high sensitivity, the presence of structurally similar glucocorticoids during testing can cause cross-reactivity, leading to erroneous positive judgments [15,16,17].

In recent years, electrochemical sensors have demonstrated broad application prospects for cortisol detection [18]. Among them, cortisol detection is achieved by measuring the electrical signals generated during the redox reaction between cortisol and the modified materials on the sensing interface [19]. In the oxidation of cortisol, the β-hydroxyl group is oxidized to a carbonyl group [20,21,22]. For the reduction reaction, the electrochemical signal originates from the electrocatalytic reduction of the C3 position of cortisol [23,24,25,26,27]. For example, Hassan et al. [21] reported an electrocatalytic sensor featuring a polyaniline-graphene oxide composite modified on a glassy carbon electrode for cortisol detection, based on the oxidation of cortisol (e.g., the conversion of its β-hydroxyl group to a ketone). The sensor has a linear range (100 μM–10 mM) and a limit of detection (30 μM). In addition, Goyal et al. [25] explored the electrocatalytic reduction of cortisol on edge-plane pyrolytic graphite electrodes, determined a detection range (100–2000 nM), and a limit of detection of 88 nM via square-wave voltammetry. However, cortisol exhibits different physiological concentrations depending on the biofluid type: 140–690 nM in serum [28], 3.6–8.3 nM in saliva [29], and 22.5–391 nM in sweat [30]. Although the aforementioned methods have been applied to the electrochemical detection of cortisol, their detection ranges and sensitivity are not satisfactory, which would affect the accuracy of rapid detection in actual samples. Therefore, it is urgent to develop a novel detection technology for rapid, high-sensitivity detection in related fields, with excellent performance, simple operation, low cost, and fast response time.

Thanks to their excellent electrochemical performance, carbon-based materials have shown great promise for applications in the field of electrochemical analysis and are widely used in the construction of electrochemical sensors [31]. Graphene, with its outstanding conductivity and large specific surface area, is regarded as an electrode material with great application potential [25,32]. Among these, laser-induced graphene (LIG) with a three-dimensional (3D) porous microstructure has a large specific surface area, giving it significant advantages in electrocatalysis [33,34,35]. The LIG electrode can be prepared simply and efficiently by laser scanning thermoplastic polymer substrates such as polyimide (PI) [36]. These electrodes are inexpensive, stable, and portable, making them suitable for practical applications. However, single LIG devices typically exhibit limited sensitivity [37,38]. Noble metal nanoparticles such as platinum, silver, and gold can be modified to enhance sensors’ sensitivity [39,40,41]. For example, our group immobilized platinum nanoparticles on an LIG-based electrode (LIG/Pt) to construct an electrochemical sensor for the rapid and sensitive detection of carbendazim [42].

**Scheme 1:**
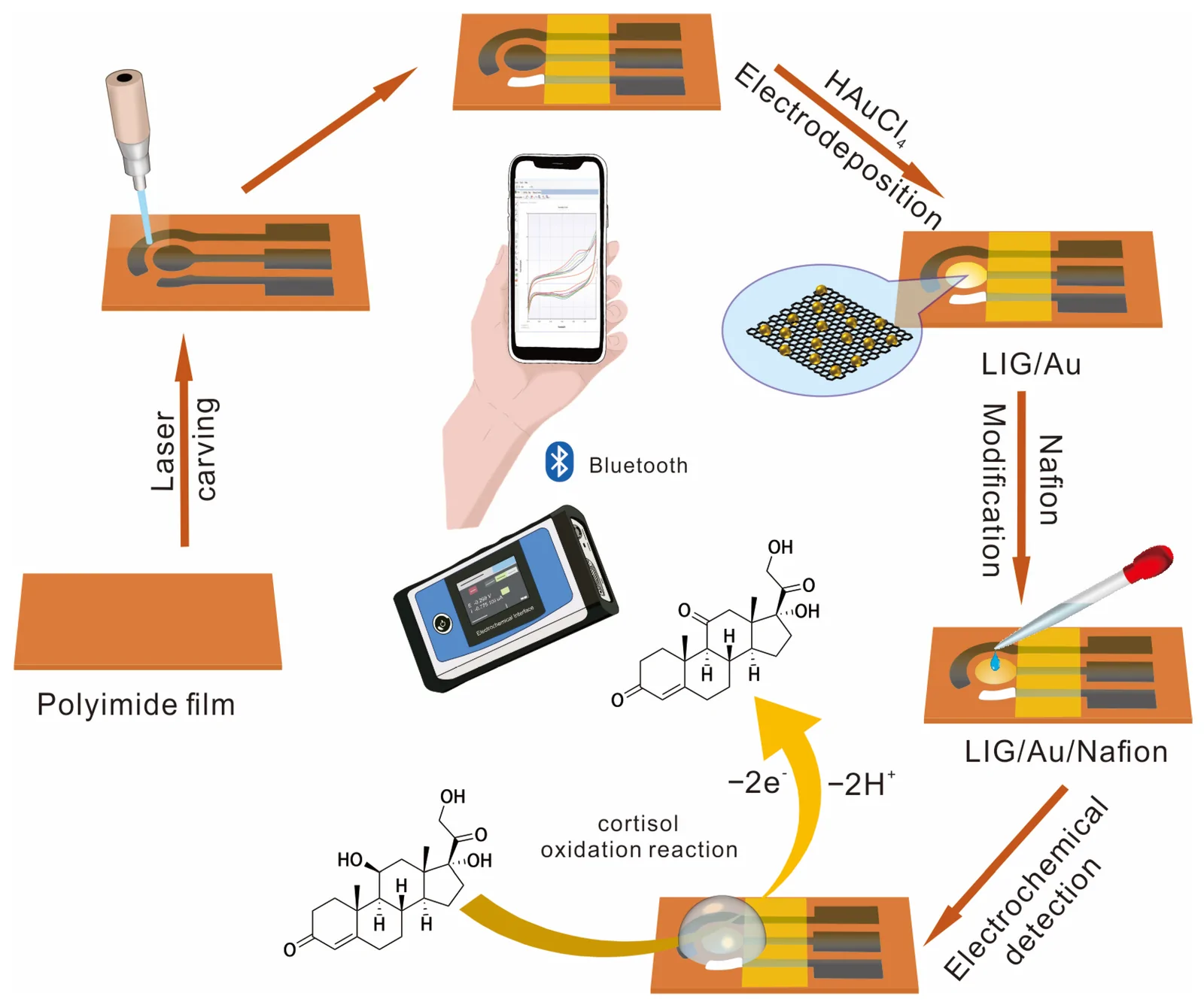
Preparation of LIG/Au/Nafion sensor and electrochemical detection of cortisol.

Inspired by this, a novel electrocatalytic nanosensing interface based on LIG was developed for the rapid detection of cortisol. In this sensing system, an LIG electrode was first fabricated on a PI film using laser printing technology. Subsequently, the gold nanoparticles (AuNPs) were deposited onto the LIG surface to electrocatalytically oxidize cortisol and enhance interfacial conductivity, followed by Nafion modification to enhance the electrocatalytic oxidation of cortisol. Finally, the resulting electrochemical signal can be accurately detected by an electrochemical workstation. The complete preparation process and sensing mechanism are illustrated in Scheme 1.

## 2. Materials and Methods

### 2.1. Reagents and Instruments

The chemicals used in this study, including cortisol (>98%), glucose, lactic acid, ascorbic acid, NaCl, KCl, NaOH, and HAuCl_4_·3H_2_O, were all supplied by Aladdin Biochemical Technology Co., Ltd., Shanghai, China. Urea was supplied by Macklin Biochemical Technology Co., Ltd., Shanghai, China. Corticosterone (99.4%) was supplied by ANPEL-TRACE Standard Technical Services Co., Ltd., Shanghai, China. K_3_Fe(CN)_6_ and K_4_Fe(CN)_6_ were purchased from Sinopharm Chemical Reagent Co., Ltd., Shanghai, China. PBS (0.01 M, pH 7.4) was supplied by Yuanye Biochemical Technology Co., Ltd., Shanghai, China. Chemical reagents of analytical grade purity are used in this study. PI film (80 μm thick), PI tape (24 μm thick), and Nafion (5%) solution were supplied by DuPont, Wilmington, DE, USA. The Ag/AgCl paste (E2414) was supplied by Elcon in Wareham, MA, USA.

Synthesis of the LIG on PI substrates was carried out with a computer-interfaced laser printing micromachine from Jiayin Nanotechnology Co., Ltd. (5.5 W, Tianjin, China). The morphological characteristics of various electrodes were characterized via a field-emission scanning electron microscope (SEM, HITACHI SU8010, Hitachi, Ltd., Tokyo, Japan). Crystal structure analysis was elucidated using an X-ray diffractometer (XRD, ESCALAB 250, Waltham, MA, USA). Raman spectroscopy characterization of the sensing interface was conducted using a Raman spectroscopy system (LabRAM HR Evolution, Horiba Jobin Yvon, Kyoto, Japan). HELA Sweat Collector (SWI-007, Nanjing, China) was applied for sweat collection. All electrochemical measurements were conducted using a handheld electrochemical workstation (PalmSens4, PalmSens BV, Houten, The Netherlands).

### 2.2. Preparation of the LIG/Au/Nafion Sensor

The PI film was ultrasonicated in ethanol for 5 min, followed by drying with nitrogen. 3D porous graphene was fabricated on the PI substrate using a laser power of 2.64 W at a scanning speed of 1.175 cm/s. The reference electrode area was then coated with Ag/AgCl paste and dried at 70 °C for 1 h. After encapsulation with PI tape, AuNPs were electrodeposited onto the surface of LIG by the chronoamperometric (CA) method at the potential of −1.4 V for 120 s in a 25 mM HAuCl_4_ solution. Subsequently, the LIG/Au electrode was slowly rinsed using deionized water. Finally, 6 μL of 0.1% Nafion solution was drop-cast onto the working electrode and dried at 35 °C for 1 h to produce the LIG/Au/Nafion sensor.

### 2.3. Electrochemical Detection of Cortisol

The LIG/Au/Nafion sensor was connected to a hand-held electrochemical workstation, and cortisol detection was conducted via the CV method. A 50 μL cortisol standard solution in 0.01 M PBS was dropped onto the sensor surface. CV analysis was performed using an initial pretreatment at −0.3 V for 60 s, followed by a potential scan (−0.1 V~0.5 V, 0.02 V/s). The CV method was used for the detection of cortisol in this experiment.

### 2.4. Analysis of Artificial Sweat

In this study, artificial sweat (AS) was formulated in accordance with the reported protocol [43,44]. AS was prepared in PBS (0.01 M, pH 7.4) containing 85 mM NaCl, 13 mM KCl, 17 mM lactic acid, and 16 mM urea, and then the pH was adjusted to 7.0 with NaOH (0.1 M). To evaluate the sensor’s detection performance in physiological conditions (22.1–391.5 nM) [45], a 500 μM cortisol stock solution was diluted in the AS by gradient dilutions. Subsequently, cortisol solutions with concentrations (20, 50, 100, 200, and 500 nM) were prepared within the AS and were stored at 4 °C.

### 2.5. Cortisol Determination in Sweat Sample

Sweat samples were collected from candidate volunteers using the HELA sweat collector. The samples were collected in a centrifuge tube and stored at 4 °C to prevent any chemical degradation. The samples were then thawed to 27 °C, centrifuged, and diluted 1:1 with 0.01 M PBS. Cortisol samples at different concentrations (20 nM, 50 nM, and 100 nM) were then prepared using a spiking method for analytical testing.

## 3. Results and Discussion

### 3.1. Morphological and Structural Characterization

**Figure 1:**
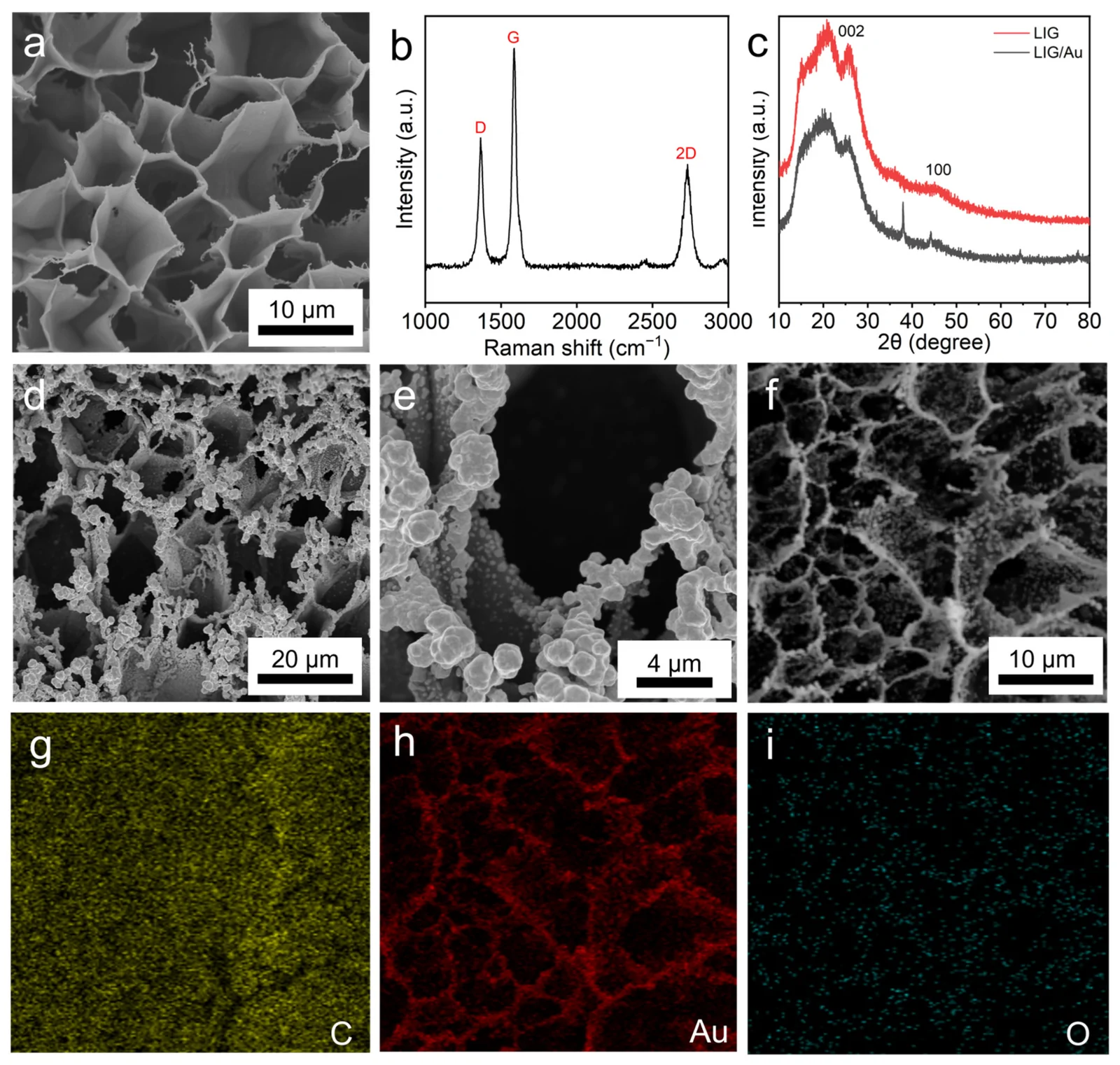
Characterization of the prepared LIG-based electrodes. (**a**) SEM image of the LIG. (**b**) The Raman spectrum of the LIG. (**c**) The XRD analysis of the LIG and LIG/Au. (**d**,**e**) SEM image of the LIG/Au. (**f**–**i**) EDS images of LIG/Au/Nafion.

The microstructures of different electrode interfaces (the LIG, LIG/Au, and LIG/Au/Nafion) were characterized. As shown in Figure 1a, the LIG exhibits a 3D porous structure, which can provide a large number of active sites for the catalytic reaction of cortisol. Furthermore, Raman spectroscopy is a classic characterization technique for carbon materials, exhibiting extremely high sensitivity to the internal geometric structure and chemical bonding states of molecules. As displayed in Figure 1b, the LIG exhibits three characteristic Raman peaks: 1366 cm^−1^ is the characteristic peak for the D band, 1587 cm^−1^ is the peak for the G band, and 2725 cm^−1^ corresponds to the 2D band. Structural defects within graphene are reflected by the D peak, while the G peak is closely related to the E2g vibrational mode characteristic of graphitic carbon materials. The distinct 2D peak indicates that the graphene component was detected in the material. The ID/IG intensity ratio is only 0.58, indicating that the graphene has a multilayer graphene structure [46,47,48,49,50].

The XRD pattern (Figure 1c) shows the comparative analysis results between LIG and the LIG/Au electrode. The LIG exhibits a low-intensity, broad graphene characteristic peak (002) at 2θ = 25.5° and a weak (100) diffraction peak at 45.7° [51,52]. Combined with Raman spectra, these experimental results indicate that the prepared LIG has undergone effective graphitization transformation and possesses a high-quality graphene crystal structure. Additionally, the LIG/Au electrode also displayed diffraction peaks characteristic of face-centered cubic gold crystals at 37.92°, 44.22°, 64.37°, and 77.36° [53]. Combined with the SEM images (Figure 1d,e), the AuNPs prepared by electrodeposition exhibit a cauliflower-shaped morphology, and the average particle size of AuNPs measured by ImageJ 1.51n software is 148.3 nm.

CV measurements were performed at various scan rates (20–120 mV·s^−1^), and the anodic peak currents (*I*_pa_) were plotted against *ν*^1/2^ (Figure S1). The electroactive surface area of the modified LIG was calculated based on the Randles–Sevcik equation (*I*_pa_ = 2.69 × 10^5^*A D*^1/2^ *n*^3/2^ *ν*^1/2^ *C*) [54,55], where *A* is the electroactive surface area of the modified electrode (cm^2^), *D* is the diffusion coefficient of the molecule in the [Fe(CN)_6_]^3−/4−^ solution (7.60 × 10^−6^ cm^2^·s^−1^), *n* is the number of electrons involved in the redox reaction (*n* = 1), *ν* is the scan rate (V·s^−1^), and *C* is the concentration (5.0 mM). Since *n*, *D*, and *C* are all constants, *A* is directly proportional to the slope of the curve plotted against *ν*^1/2^ for the anodic peak current *I*_pa_. The electroactive surface areas of LIG and LIG/Au are 0.173 cm^2^ and 0.213 cm^2^, respectively. The specific surface area of LIG/Au is 1.23 times that of LIG, which further demonstrates that AuNP modification can increase the electrode’s specific surface area [56].

The results of the element distribution are shown in Figure 1f–i and Figure S2a, clearly revealing the presence of Au elements loaded on the graphite carbon surface [34]. In addition, the elemental distribution maps also reveal the presence of F, O, and S elements, all of which are characteristic components of Nafion (F originates from its C–F backbone, while O and S derive from the sulfonic acid groups) [57]. Therefore, the concurrent detection of F, O, S, and Au fully confirms that Nafion has been successfully modified onto the electrode interface, and that the AuNPs are stably loaded on the graphene surface.

### 3.2. Electrochemical Characterization

These fabricated electrodes were characterized using the CV method in a 1.0 mM [Fe(CN)_6_]^3−/4−^ solution containing 0.1 M KCl with a potential range from −0.3 V to +0.4 V. As shown in Figure S2b, the bare LIG electrode clearly exhibited two redox peaks. After electrodeposition of AuNPs, a significant enhancement in peak response was observed, and the peak current increased by 9.02 μA compared to the LIG electrode. This enhanced response stems from the excellent electrical conductivity of the AuNPs [10,53,58], and after modification with Nafion, the redox peak response showed a significant decrease. This decrease was attributed to the formation of a non-conductive Nafion film on the electrode surface, which hindered the diffusion and migration of ions. Furthermore, the negatively charged sulfonic acid groups in the Nafion matrix exerted electrostatic repulsion on the anions, further hindering the charge transfer process [51,57,59]. Combined results from morphological, structural, and electrochemical characterizations confirm that AuNPs and Nafion have been successfully modified on the surface of the LIG.

### 3.3. Electrochemical Behavior of Cortisol

**Figure 2:**
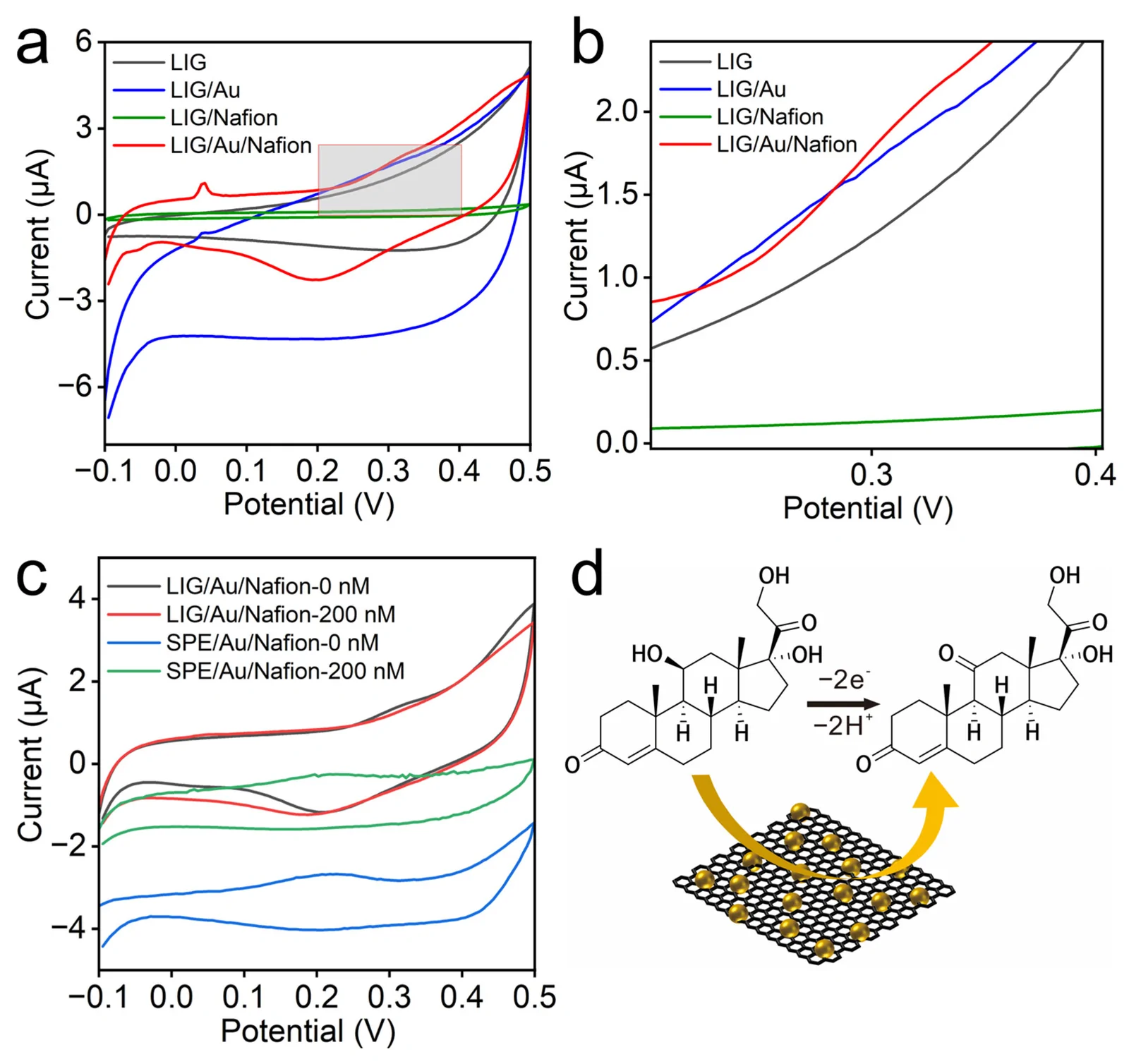
Electrochemical behavior of cortisol. (**a**) CV curves and (**b**) their enlarged views of LIG, LIG/Nafion, LIG/Au, and LIG/Au/Nafion electrodes in 200 nM cortisol solution. (**c**) CV curves of the LIG/Au/Nafion and SPE/Au/Nafion sensors with/without the addition of 200 nM cortisol. (**d**) The electrochemical catalytic mechanism of cortisol.

The electrocatalytic performances of different LIG-based electrodes were evaluated in a 200 nM cortisol solution using the CV method. As shown in Figure 2a,b, the LIG/Au/Nafion interface exhibits a higher electrocatalytic response at +0.3 V for cortisol, compared to the LIG/Au interface, while no corresponding oxidation peak was observed at the LIG/Nafion and LIG electrode interfaces. These results indicate that the introduction of AuNPs catalyzes the oxidative dehydrogenation of cortisol. And there is a synergistic effect between AuNPs and Nafion, which is attributed to the sulfonate-rich interfacial microenvironment of Nafion that facilitates the interfacial conduction and removal of the protons liberated during cortisol dehydrogenation [60,61,62].

To further confirm the electrocatalytic activity of this sensor toward cortisol, the electrochemical behaviors of the LIG/Au/Nafion sensor were investigated in a PBS solution with/without 200 nM cortisol. The results showed that an oxidation peak appeared in the cortisol solution compared to the PBS solution (Figure 2c). In addition, we further compared and analyzed the response characteristics of the sensor in cortisol detection by taking the screen-printed electrode as a reference. This indicates that the LIG/Au/Nafion sensor has a good detection effect on cortisol. Based on the relevant research [20,21,22,27], it can be inferred that the β-hydroxyl group of cortisol undergoes an oxidation reaction, losing two electrons to form a carbonyl group. The corresponding oxidation reaction mechanism is shown in Figure 2d.

### 3.4. Optimization of Experimental Conditions

**Figure 3:**
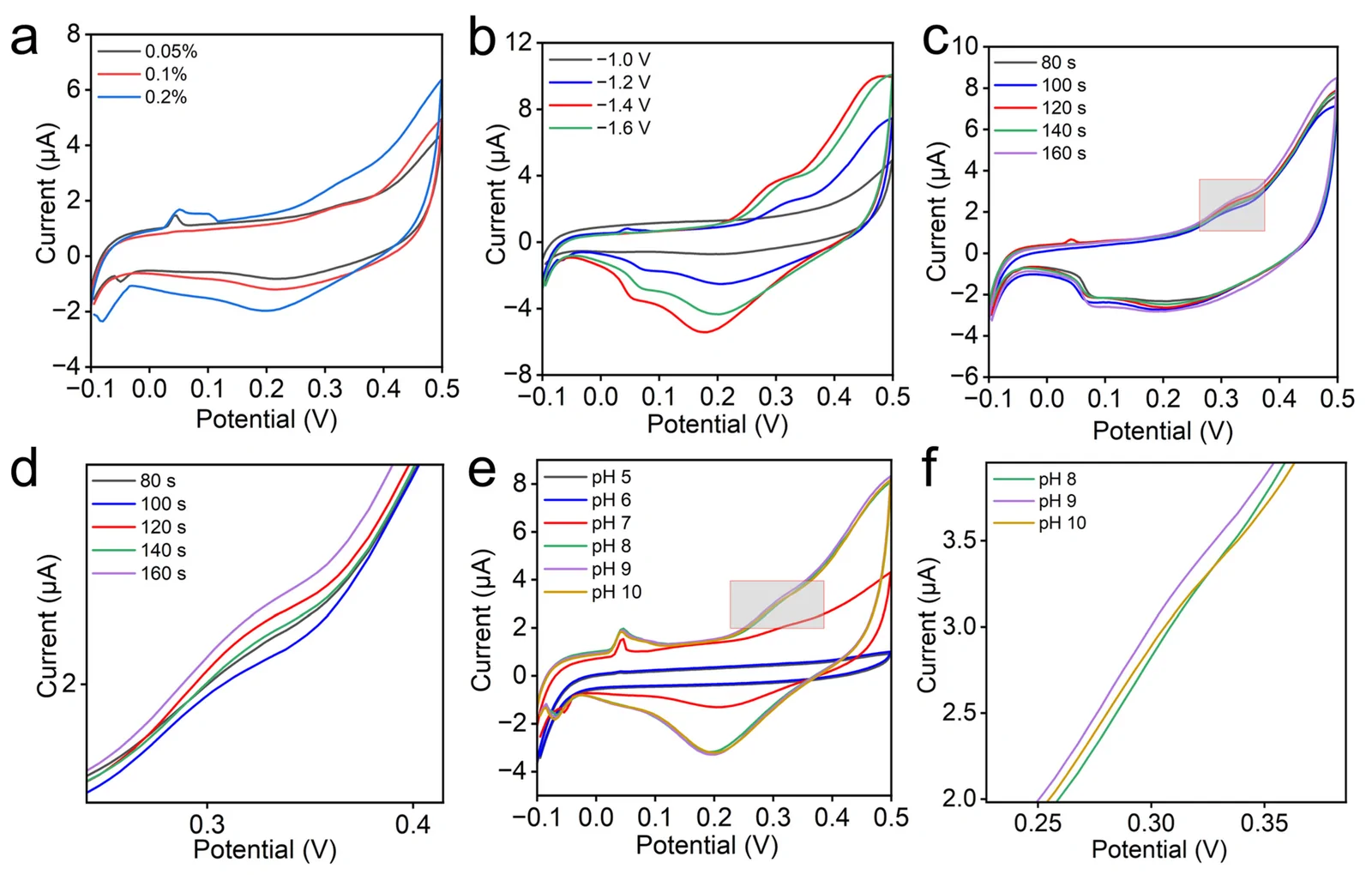
Optimization of sensor preparation conditions. CV responses of LIG/Au/Nafion sensors in 200 nM cortisol solution under (**a**) different Nafion concentrations, (**b**) electrodeposition potential, (**c**) electrodeposition time for AuNPs, and (**e**) pH conditions; (**d**,**f**) the enlarged images of Figure 3c,e.

The concentration of Nafion solution directly affects the thickness and pore size of the Nafion film, which indirectly influences the electrocatalytic activity of the LIG/Au/Nafion sensor toward cortisol. Therefore, Nafion solutions with various concentrations (0.05%, 0.1%, and 0.2%) were optimized. The experimental data revealed that when the concentration of Nafion solution was increased from 0.05% to 0.1%, the peak height of the oxidation peak increased accordingly. This phenomenon may be due to the more uniform film formed by the 0.1% Nafion solution (Figure 3a). However, when the concentration was increased to 0.2%, the peak height of cortisol decreased. A possible reason is that the increased thickness of the Nafion film hinders the migration of cortisol to the catalytically active sites [57,59,63]. Therefore, this study confirmed that 0.1% is the optimal concentration of Nafion for subsequent experiments.

Under the optimal Nafion concentration, we further optimized parameters including the electrodeposition potential and deposition time of AuNPs. As shown in Figure 3b, a series of LIG/Au electrodes for cortisol detection were prepared by varying the deposition potential. A comparison of the oxidation peak heights revealed that the strongest peak was observed at a deposition potential of −1.4 V, indicating that AuNPs prepared at this potential, combined with 0.1% Nafion, exhibit the best electrocatalytic activity toward cortisol.

Furthermore, the effect of electrodeposition time on the sensor’s detection performance was investigated (Figure 3c,d). The experimental results show that the peak height of the oxidation peak was highest at a deposition time of 120 s. Insufficient deposition time may reduce the number of catalytic sites; excessively long deposition time causes aggregation of AuNPs and a decrease in specific surface area, thereby lowering the sensor sensitivity [38,40]. In summary, the optimal electrodeposition parameters for preparing the LIG/Au/Nafion sensor were determined to be an electrodeposition potential of −1.4 V and a time of 120 s.

Finally, we studied the effect of the pH of the electrolyte solution on the LIG/Au/Nafion sensor (Figure 3e,f). Results showed that the oxidation peak current of cortisol gradually increased with the pH from 5 to 10, reaching the maximum value at pH 10. This may be due to alkaline conditions promoting the deprotonation of cortisol, thereby enhancing its oxidation response. In order to match the near-neutral physiological environment and thereby minimize sample pretreatment, we chose the pH value of the electrolyte solution at 7.4.

### 3.5. Analytical Performance for Cortisol Detection

**Figure 4:**
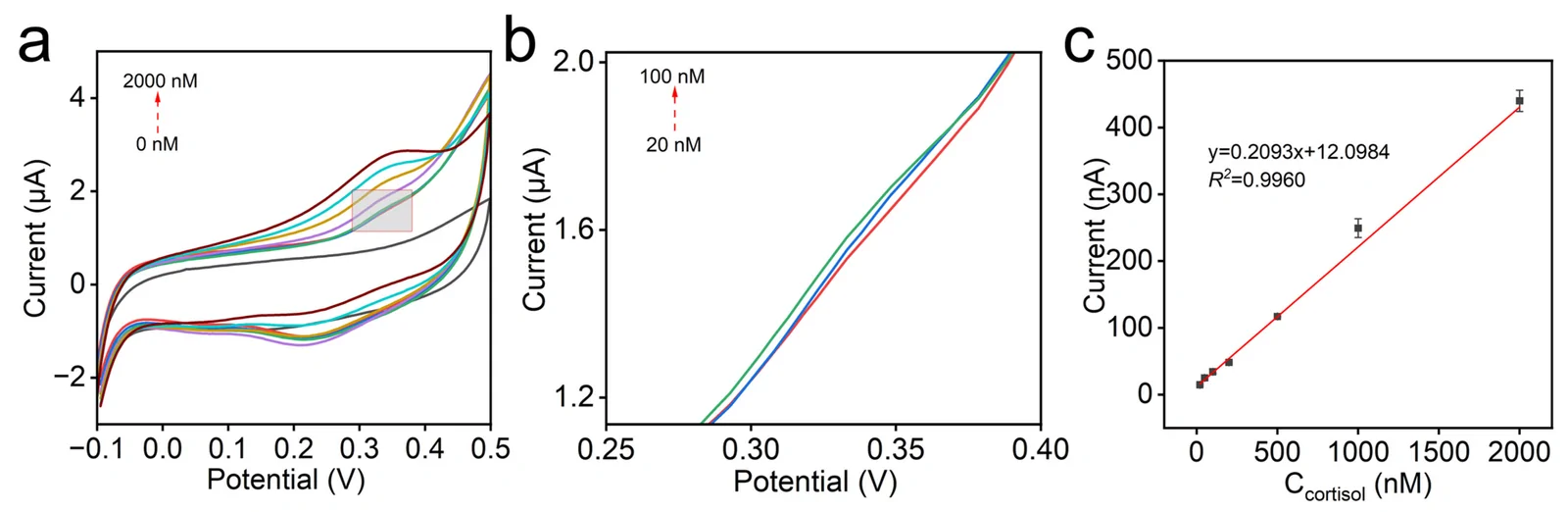
CV response of the (**a**) original and (**b**) enlarged image of the LIG/Au/Nafion sensor in cortisol solution with concentrations of 0 to 2000 nM. (**c**) Corresponding calibration curve showing the relationship between cortisol concentration and CV peak height.

Under optimal experimental conditions, the electrocatalytic response of the sensor in different cortisol solutions (20, 50, 100, 200, 500, 1000, and 2000 nM) was investigated using the CV method. As shown in Figure 4a,b, an oxidation peak was observed, and its response increased with increasing cortisol concentration. As shown in Figure 4c, there is a linear relationship between cortisol concentration (20 to 2000 nM) and peak height. As defined, the detection limit (LOD = 3 Sb/m), with Sb representing the standard deviation of CV in the blank experiment and m as the slope of the calibration curve [42]. The LOD of this sensor was calculated to be 1.02 nM (i.e., 0.369 ng/mL), which is below the physiological concentration range of cortisol in human sweat [45].

In addition, the performance of the LIG/Au/Nafion sensor prepared in this study was compared with that of previously reported cortisol sensors based on the electrocatalytic method (Table S1). The LIG/Au/Nafion sensor developed in this study can detect cortisol with lower oxidation potential and high sensitivity in PBS electrolyte instead of KOH, H_2_SO_4_, etc., indicating its stronger biocompatibility and practical value.

### 3.6. Selectivity, Reproducibility, and Stability Studies of LIG/Au/Nafion Sensor

**Figure 5:**
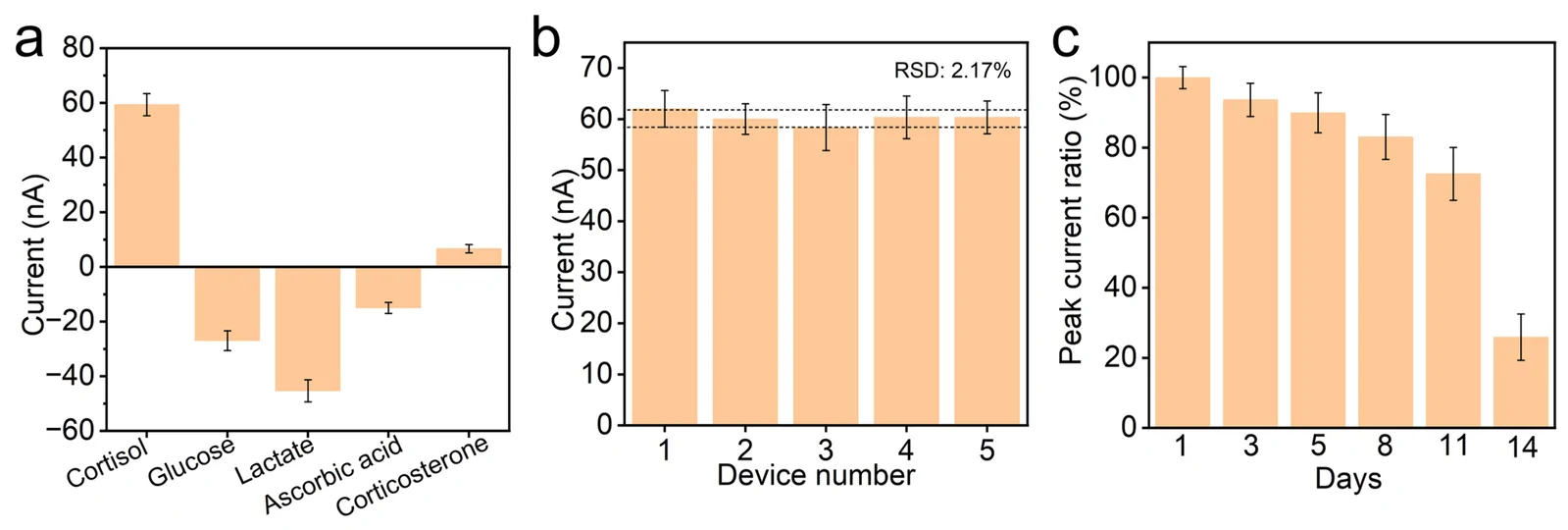
Evaluation of the (**a**) selectivity, (**b**) reproducibility, and (**c**) stability of the LIG/Au/Nafion sensor.

The selectivity of the LIG/Au/Nafion sensor was tested in various sample solutions, including potential interferents (glucose, lactate, ascorbic acid (2 μM)) and corticosterone (200 nM). The sensor showed no evident oxidation peak for glucose, lactic acid, and ascorbic acid, and the oxidation peak of corticosterone was barely detectable (Figure 5a), indicating its favorable selectivity.

In addition, the reproducibility of the sensor was evaluated by testing the peak height of five LIG/Au/Nafion sensors for cortisol at a concentration of 200 nM. The current responses of the five sensors showed minimal variation (Figure 5b), with a relative standard deviation not exceeding 2.17%, indicating that the sensor exhibits good reproducibility. Furthermore, the storage stability of the LIG/Au/Nafion sensor was investigated by testing its response to cortisol (200 nM) in 4 °C storage under N_2_ for five days. From Figure 5c, the peak height response retained about 93.63%, 89.93%, 83.07%, and 72.5% of its initial value after 3, 5, 8, and 11 days, respectively. On day 14, the response remained at only 25.9% of the initial value. This decrease could be attributed to oxidation of the sensing interface during storage.

### 3.7. Real Sample Analysis in AS and Sweat

**Figure 6:**
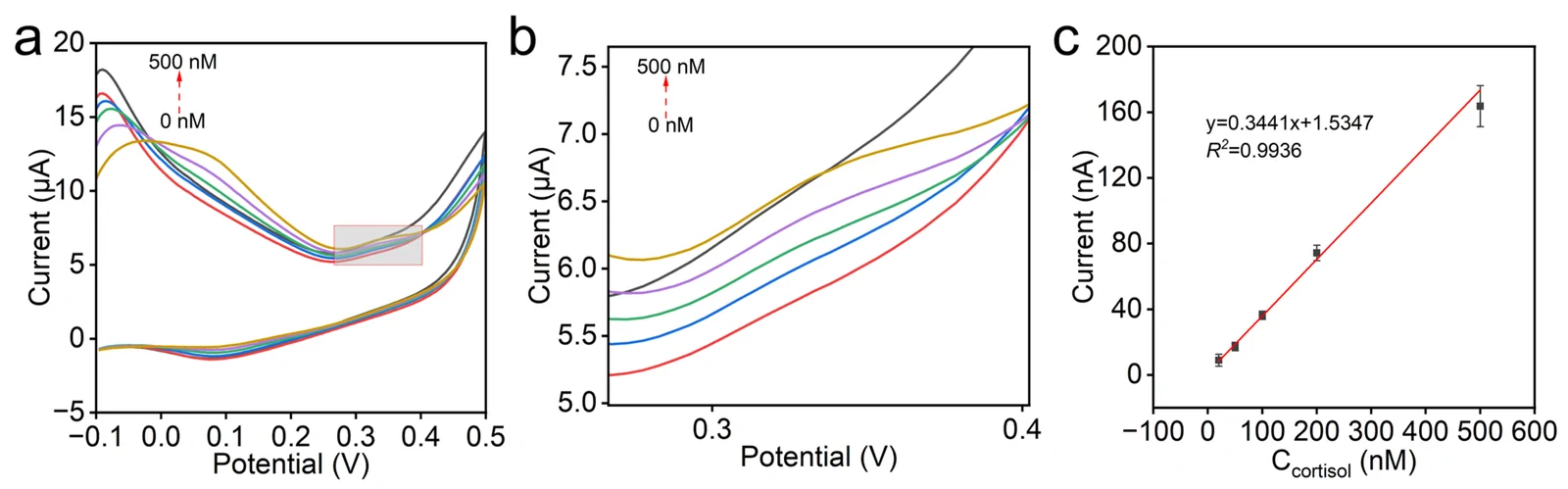
CV response of the (**a**) original and (**b**) enlarged image of the LIG/Au/Nafion sensor to cortisol (20–500 nM) in AS. (**c**) Corresponding calibration curve showing the relationship between cortisol concentration (20–500 nM) and CV peak height.

The electrochemical response was evaluated in AS. At a potential of +0.33 V, a slight increase in peak height was observed as the cortisol concentration increased (Figure 6a,b). Compared to the measurement of cortisol in PBS solution, the peak height response generated by AS was relatively low, and the oxidation peak shifted positively to 0.33 V, which indicates that the AS may affect the electron transfer rate at the interface [64,65]. As shown in Figure 6c, a good linear relationship between the concentration and peak height value was observed (ranging from 20 nM to 500 nM in AS). The above results indicated that this sensor is both feasible and accurate for detecting complex mechanisms.

In this study, sweat samples were collected using a HELA sweat collector. Before analysis, no cortisol was detected in these samples. Recovery of cortisol was evaluated at three spiking levels (20, 50, and 100 nM) in blank sweat samples. The spiked cortisol was detected by the prepared sensor and ELISA. Before detection, the samples were diluted 1:1 with PBS buffer. As shown in Table 1, the average recovery rates of cortisol detected by the prepared sensor and ELISA range from 96.76% to 113.14% and 97.03% to 104.61%, respectively, indicating the feasibility of our sensor in real sample analysis.

## 4. Conclusions

In this study, a composite sensing interface based on AuNPs and Nafion was prepared on the surface of the LIG electrode. The electrocatalytic oxidation mechanism of the LIG/Au/Nafion composite interface toward cortisol was elucidated, and the electrocatalytic sensing method for cortisol detection was established. Under optimal experimental conditions, the obtained LIG/Au/Nafion sensor exhibited a linear range of 20 nM to 2000 nM for cortisol in PBS solution, with a correlation coefficient (*R*^2^) of 0.9960 and an LOD of 1.02 nM. In addition, the sensor exhibited a linear range of 20 nM to 500 nM for cortisol in AS solution and showed a favorable recovery rate in spiked sweat samples. Compared with other sensors for electrocatalytic sensing of cortisol, this method is sensitive in neutral media rather than harsh acids/bases, which can offer a reliable strategy for cortisol detection in real sweat analysis.

## Figures and Tables

**Table 1:** Cortisol analysis in sweat spiked samples by LIG/Au/Nafion sensor and ELISA method (*n* = 3).

Method	Added (nM)	Found (nM)	Recovery (%)	RSD (%, *n* = 3)
	20	19.35 ± 2.99	96.76	15.45
LIG/Au/Nafion	50	51.28 ± 7.48	102.56	14.59
	100	113.14 ± 4.56	113.14	4.03
	20	20.71 ± 0.99	103.53	4.78
ELISA	50	48.51 ± 3.70	97.03	7.62
	100	104.61 ± 3.76	104.61	3.59

## Data Availability

The data used to support the findings of this study can be made available by the corresponding author upon request.

## Supplementary Materials

The following supporting information can be downloaded at: https://www.mdpi.com/article/10.3390/bios16100562/s1, Figure S1: CV curves of (a) LIG and (c) LIG/Au electrodes in 0.1 M KCl with 5.0 mM K_3_[Fe(CN)_6_] at scan rates from 20 to 120 mV·s^−1^. The corresponding plot of anodic peak currents (*I*pa) against varying scan rates of (b) LIG and (d) LIG/Au electrodes; Figure S2: (a) EDS characterization image of the LIG/Au/Nafion sensor. (b) CV curves of the LIG-based electrodes in a 1.0 mM K_3_[Fe(CN)_6_] solution containing 0.1 M KCl; Figure S3: Measurement of (a) selectivity and (b) its enlarged image, (c) stability, and (d) reproducibility of the LIG/Au/Nafion sensor; Table S1: Comparison of sensors for electrocatalytic redox of cortisol.
