# Supplementary material for: Electrochemical Sensor Based on Laser-Induced Graphene for Cortisol Detection in Physiological Media
Source: Biosensors (Basel). 2026 Oct 5;16(10):562. doi: 10.3390/bios16100562 (PMC13702165; doi:10.3390/bios16100562)
Supplement: Supplementary file 1 [file biosensors-16-00562-s001.zip › biosensors-4529122-supplementary.pdf]

Supplementary Information

Electrochemical Sensor Based on Laser-Induced Graphene for Cortisol Detection in Physiological Media

Shuai Zhang [1,2,†], Chao Zhang [1,†], Jingwei Zhang [2], Yuran Wang [2], Yingze Sun [1], Yijiang Zhou [2], and Fengnian Zhao [2,*]

1 Information Technology Research Center, Chinese Academy of Fishery Sciences, Beijing 100141, China; zhangchao@cafs.ac.cn (C.Z.); sunyz@cafs.ac.cn (Y.S.)
2 School of Light Industry Science and Engineering, Beijing Technology and Business University, Beijing 102488, China; 2431042212@st.btbu.edu.cn (S.Z.); 2531042282@st.btbu.edu.cn (J.Z.); 2531041104@st.btbu.edu.cn (Y.W.); beijing20300302@163.com (Y.Z.)
* Correspondence: zhaofn@btbu.edu.cn
† Shuai Zhang and Chao Zhang contributed equally to this work.

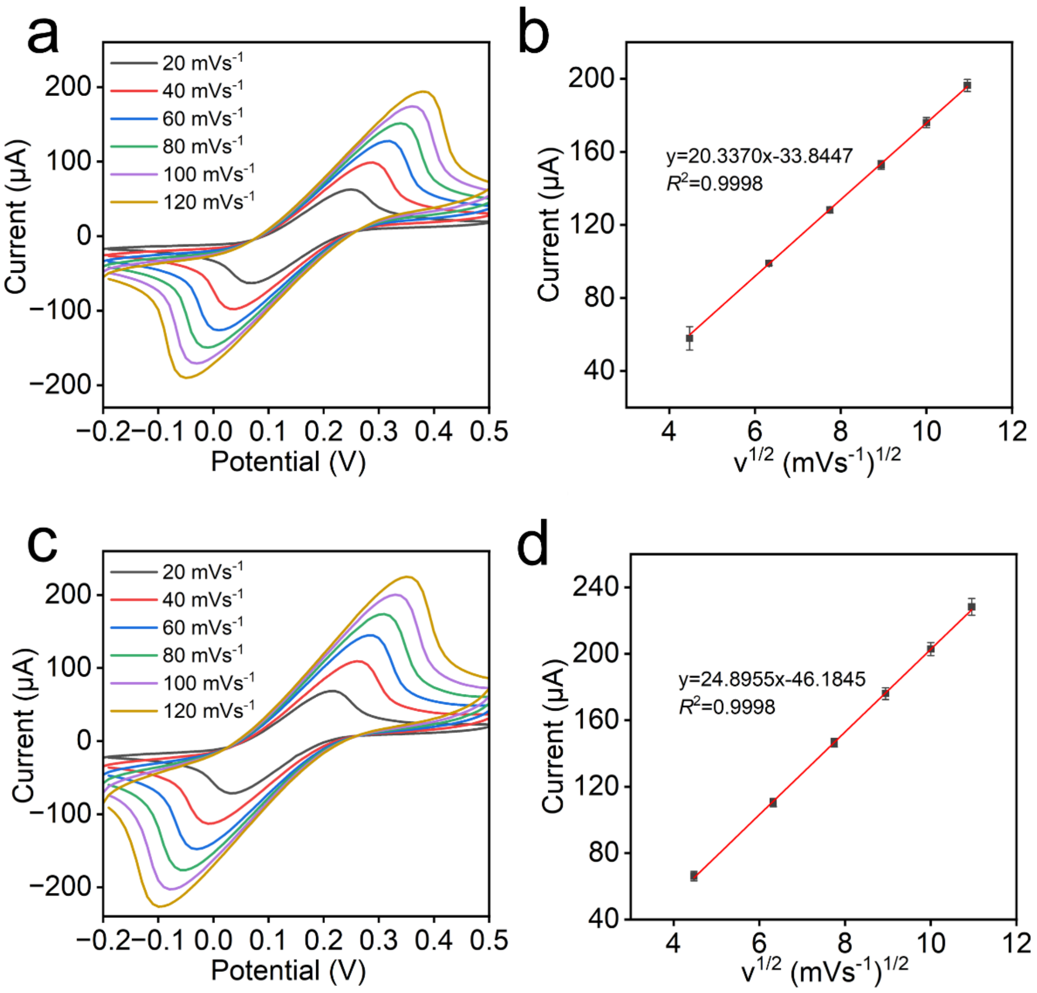

Figure S1. CV curves of (a) LIG and (c) LIG/Au electrodes in 0.1 M KCl with 5.0 mM $K_3[Fe(CN)_6]$ at scan rates from 20 to 120 mV·s^{-1}. The corresponding plot of anodic peak currents (*I*pa) against varying scan rates of (b) LIG and (d) LIG/Au electrodes.

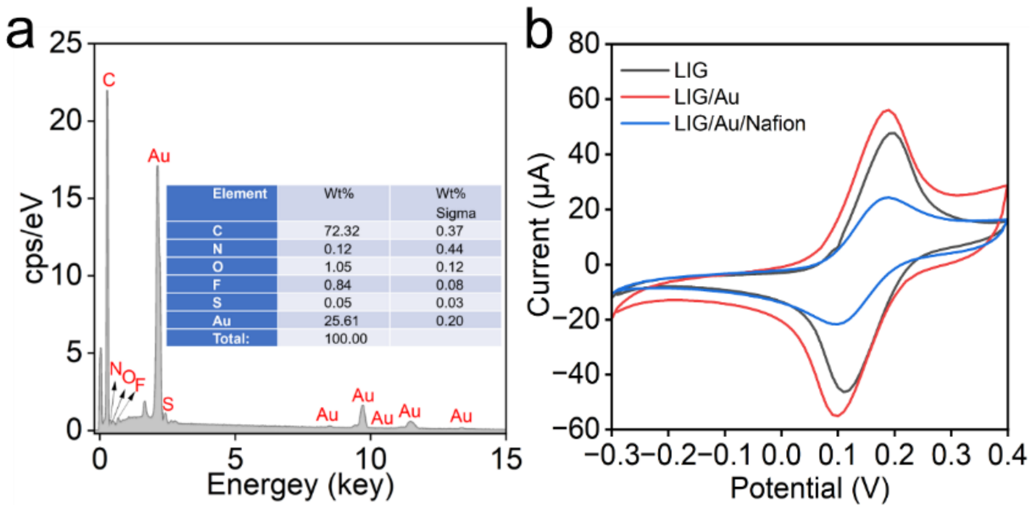

Figure S2. (a) EDS characterization image of the LIG/Au/Nafion sensor. (b) CV curves of the LIG-based electrodes in a 1.0 mM $K_3[Fe(CN)_6]$ solution containing 0.1 M KCl.

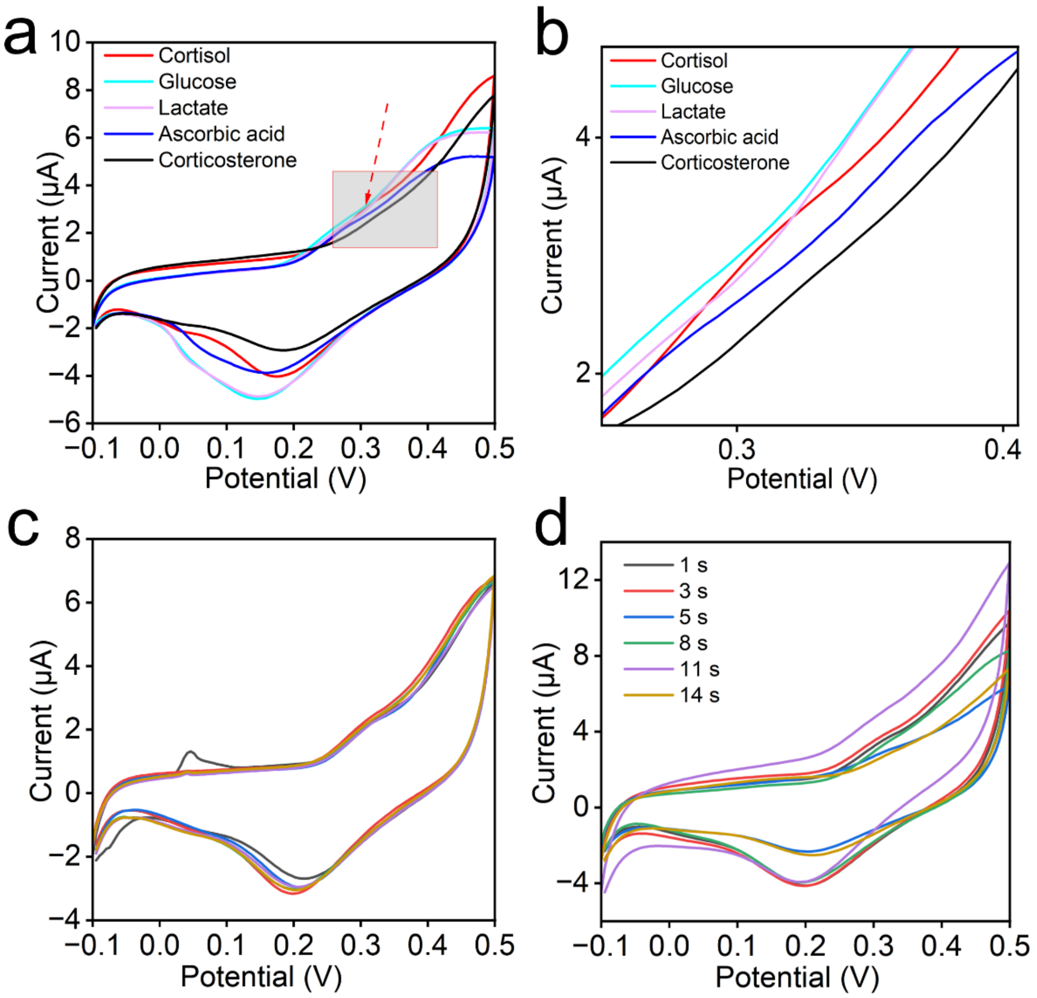

Figure S3. Measurement of (a) selectivity and (b) its enlarged image, (c) stability, and (d) reproducibility of the LIG/Au/Nafion sensor.

Table S1. Comparison of sensors for electrocatalytic redox of cortisol.

Sensor	Method	Redox	Potential	Electrolyte	Sensitivity	Linear Range	LOD	Ref
GCE/CoO/Nafion	DPV	Reduction	-0.5 V	NaOH	/	1–1000 nM<br>1–9 μM	0.49 nM	[1]
EPPGE	SWV	Reduction	-1.33 V	PBS	45 $nAnM^{-1}$	100–2000 nM	88 nM	[2]
PGE/GR/ZnO	DPV	Reduction	-0.58 V	PBS	/	0.5–11.5 nM	0.15 nM	[3]
CFME	FSCV	Reduction	-1.0 V	PBS	0.87 $nA\mu M^{-1}$	10–40 μM	/	[4]
SPCE/MWCNTs-CuTPP	DPV	Reduction	-1.26 V	PBS	/	50 fM–100 nM	50 fM	[5]
GCE/PANI-GO	DPV	Oxidation	0.29 V	PBS	/	0.1–10 mM	30 μM	[6]
SPE/Ni	CV<br>CA	Oxidation	0.6 V	KOH	/	0.25–25 μM	74.0 nM	[7]
SPE/Pd/Au/Nafion	CV	Oxidation	1.1 V	H_2SO_4	/	5 pM–5 mM	/	[8]
LIG/Au/Nafion	CV	Oxidation	0.3 V	PBS	217 $nA\mu M^{-1}$	20–2000 nM	1.02 nM	This work

Notes: GCE, Glassy carbon electrode; EPPGE, Edge-plane pyrolytic graphite electrode; PGE, Pyrolytic graphite electrode; GR, Graphene; CFME, Carbon fiber microelectrodes; MWCNTs, Multi-walled carbon nanotubes; CuTPP, 5,10,15,20-tetraphenylporphyrinato copper (II); PANI-GO, Graphene Oxide-Polyaniline; SPE, Screen printed electrode; CA, Chronoamperometry.

References

1. Sharma, N.; Reddy, A.S.; Yun, K. Electrochemical Detection of Hydrocortisone Using Green-Synthesized Cobalt Oxide Nanoparticles with Nafion-Modified Glassy Carbon Electrode. *Chemosphere* **2021**, *282*, 131029. https://doi.org/10.1016/j.chemosphere.2021.131029
2. Goyal, R.N.; Chatterjee, S.; Rana, A.R.S. A Comparison of Edge- and Basal-Plane Pyrolytic Graphite Electrodes towards the Sensitive Determination of Hydrocortisone. *Talanta* **2010**, *83*, 149-155. https://doi.org/10.1016/j.talanta.2010.08.054
3. Rison, S.; Rajeev, R.; Bhat, V.S.; Mathews, A.T.; Varghese, A.; Hegde, G. Non-Enzymatic Electrochemical Determination of Salivary Cortisol Using ZnO-Graphene Nanocomposites. *RSC. Adv.* **2021**, *11*, 37877-37885. https://doi.org/10.1039/D1RA07366D
4. Hadad, M.; Hadad, N.; Zestos, A.G. Carbon Electrode Sensor for the Measurement of Cortisol with Fast-Scan Cyclic Voltammetry. *Biosensors* **2023**, *13*, 626. https://doi.org/10.3390/bios13060626
5. Manickam, P.; Fernandez, R.E.; Umasankar, Y.; Gurusamy, M.; Arizaleta, F.; Urizar, G.; Bhansali, S. Salivary cortisol analysis using metalloporphyrins and multi-walled carbon nanotubes nanocomposite functionalized electrodes. *Sensors and Actuators B: Chemical* **2018**, *274*, 47-53. https://doi.org/10.1016/j.snb.2018.07.133
6. Hassan, Z.; Alsalhi, S.A.; Drissi, N.; Abdelmohsen, S.A.M.; Sattar, J.; Tehreem, P.; Noor, H.; Alqurashi, H.; Henaish, A.M.A.; Farid, H.M.T. Graphene Nanoplatelets-Polyaniline Composite for the Detection of Cortisol. *J. Phys. Chem. Solids.* **2024**, *191*, 112031. https://doi.org/10.1016/j.jpcs.2024.112031
7. Gevaerd, A.; Watanabe, E.Y.; Belli, C.; Marcolino-Junior, L.H.; Bergamini, M.F. A Complete Lab-made Point of Care Device for Non-Immunological Electrochemical Determination of Cortisol Levels in Salivary Samples. *Sens. Actuat. B-chem.* **2021**, *332*, 129532. https://doi.org/10.1016/j.snb.2021.129532
8. Shaidarova, L.G.; Chelnokova, I.A.; Leksina, Y.A.; Khairullina, D.Y.; Budnikov, H.C. Flow-Injection Amperometric Determination of Adrenalin, Melatonin, and Cortisol on An Electrode Modified with A Gold–Palladium Binary System and A Nafion Film. *Inorg. Mater.* **2024**, *59*, 1474-1481. https://doi.org/10.1134/s0020168523140121
